# Adrenal failure followed by status epilepticus and hemolytic anemia in primary antiphospholipid syndrome

**DOI:** 10.1186/1477-9560-3-6

**Published:** 2005-04-18

**Authors:** Patrick Gerner, Michael Heldmann, Peter Borusiak, Vladimir Bures, Stefan Wirth

**Affiliations:** 1 Children's Hospital, HELIOS Klinikum Wuppertal, Witten-Herdecke University, Germany; 2 Department of Radiology, HELIOS Klinikum Wuppertal, Witten-Herdecke University, Germany

## Abstract

We report on a 14 year old boy who presented with the symptoms abdominal pain, fever and proteinuria. A hematoma in the region of the right pararenal space was diagnosed. Prothrombin time and activated partial thromboplastin time were prolonged, lupus anticoagulant and anticardiolipin antibodies were positive and serum cortisol was normal. Ten days after admission the boy suddenly suffered generalized seizures due to low serum sodium. As well, the patient developed hemolytic anemia, acute elevated liver enzymes, hematuria and increased proteinuria. At this time a second hemorrhage of the left adrenal gland was documented. Adrenal function tests revealed adrenal insufficiency. We suspected microthromboses in the adrenals and secondary bleeding and treated the boy with hydrocortisone, fludrocortisone and phenprocoumon.

**Conclusion:**

Adrenal failure is a rare complication of APS in children with only five cases reported to date. As shown in our patient, this syndrome can manifest in a diverse set of simultaneously occurring symptoms.

## Background

The antiphospholipid syndrome is characterized by clinical evidence of arterial or venous thrombosis and repeated presence of antiphospholipid autoantibodies. The disease, first described by Hughes in 1983 [1], may occur in a variety of conditions including neoplasms, infections, other autoimmune disease such as lupus erythematodes, and after administration of certain drugs. If found without any other demonstrable disease, it is termed primary antiphospholipid syndrome.

The autoantibodies, of which the most important are lupus anticoagulant and cardiolipin-antibodies, comprise a heterogeneous group which are mainly directed against complexes of anionic phospholipid with some phospholipid-binding proteins such as beta-2-glycoprotein I and human prothrombin. In a proportion of individuals the circulation of the antibodies may induce thrombosis of virtually any vein or artery. The most common are deep vein thrombosis, pulmonary, or cerebrovascular embolisms. In addition, other complications such as cardiomyopathy, hepatitis, hemolytic anemia, bleeding, vasculitis and renal failure have been reported [2-5].

The complexity of manifestations and the risk of severe complications, led us to conclude that this syndrome is an important differential diagnosis in patients with the described symptoms.

## Case Report

In September 2001, a previously healthy, Caucasian, 14-year-old boy was admitted to our hospital. Two weeks prior to his arrival, he had developed abdominal pain, a recurring fever of up to 39°C, and an intermittent cough. These symptoms were worsening and his general condition was deteriorating.

On examination he had diffuse abdominal pain located primarily in the upper abdomen, and his temperature was 38.5°C. His skin and the further physical examination was normal. Pertinent laboratory investigations are listed in Table 1. Of note, the activated partial thromboplastin time and prothrombin time were both prolonged, mild thrombopenia and leukocytosis of 14/nl were present, and C-reactive protein was elevated (6.1 mg/dl). The activity of factors II, V, VII and VIII was normal. There was no history of autoimmune diseases or coagulation disorders in his family.

**Table 1:** Expression of important laboratory findings

Variable	Day 1	Day 5	Day 10	Day 15	Day 25	Day 50	12 month	Normal range
IgG anti-cardiolipin antibody (GPL-U/ml)		44.7		21	60.2	30.7	37.7	<12
Lupus anticoagulant		positive		positive	positive	positive	positive	absent
Anti-β-2-Glycoprotein 1 (U/ml)				2	6	3	negative	<5
Anti-Phosphatidylserin (U/ml)				72.8	10.8	31.1		<15
Anti-Phosphatidylethanolamin (U/ml)				45.2	23.9	18.5		<15
Antinuclear antibodies			negative				negative	absent
ENA			negative					absent
Double strand-DNA			negative					absent
anti-adrenal-antibodies			negative					absent
Cortisol 0800 h		26	1.1	22		28		6–26
Renin activity			100					<3β
ACTH (pg/ml)			468					<50
Aldosteron (pg/l)			<10					12–125
DHEA-sulfate (g/dl)			<10					<280
Bleeding time (min)		10.55	5.30					<7
Partial thromboplastin time (s)	39	37	48	57	58	36	41	<35
Prothrombin time (%)	64	55	55	73	78	45	36	70–100
Sodium (mmol/l)	134	140	111	139	142	143	136	132–145
Potassium (mmol/l)	3.9	4.8	3.6	4.2	4.0	3.5	3.9	3.1–5.1
C-reactive Protein (mg/dl)	6.1	6.1	12.1	3.7	1.0	0.4	0.3	<0.5
Aspartate aminotransferase (U/l)	15	17	81	37	21	16	18	<15
Alanine aminotransferase (U/l)	20	18	67	69	40	24	22	<14
LDH (U/l)	201		442	309		256	181	<240
Creatinine (mg/dl)	0.7	0.7	1.0	0.7	0.8	0.93	0.3	0.6–1.3
β2-microglobulin (mg/dl)		0.18	0.27		0.19			<0.08
Hemoglobin (g/l)	13.6	11.1	7.6	10.8	11.8	12.1	12.4	11.8–16.8
Platelet count /nl	129	177	56	154	97	153	174	150–350
White cell count /nl	14.5	10.3	10.2	6.2	5.4	5.6	7.8	4.3–10.0

The initial abdominal ultrasound revealed a tumor in the right pararenal space. However, a clear anatomical relation to the adrenal gland could not be established (Figures 1 and 2- see Additional file 1 and 2). The left adrenal gland was slightly enlarged. Doppler ultrasonography showed no thromboses of abdominal vessels and serum cortisol was normal at this stage. The CT scan identified the tumor as hematoma.

Additional tests were positive for lupus anticoagulant and anti-cardiolipin antibodies. Bleeding time was prolonged to 11 minutes but all other coagulation tests eliminated the possibility of acute bleeding. In fact a repeated test of bleeding time a few days later was normal.

Under intravenous treatment with cefuroxim the patient's temperature normalized within two days. But by day five, C-reactive protein increased to 12 mg/dl, and the patient's temperature rose again so antibiotic therapy was extended by gentamycin.

Ten day after being admitted, the boy suddenly suffered generalized tonic-clonic seizures due to severe hyponatremia of 111 mmol/l. Serum transaminases raised to levels five times above normal range, and after three days hemoglobin dropped from 11.8 g/l to 7.6 g/l. Since LDH was elevated and haptoglobin was reduced, we diagnosed hemolytic anemia and transfused him with erythrocyte concentrates.

Two days before the acute onset of adrenal insufficiency, microhematuria began. Proteinuria increased to 1 g/l and β-2-microglobulin raised to 0.16 mg/dl but there were no clinical signs of urinary infection. As the C-reactive protein rose to 16.1 mg/dl, intravenous antibiotic treatment was changed to erythromycin, ceftriaxon, tobramycin and flucloxacillin. However, two blood cultures, urine culture, stool culture and liquor culture remained sterile. Furthermore, serum serological tests were negative for the following infections: borrelia-burgdorferi, HIV, hepatitis B virus, hepatitis C virus, listeria, leptospira, epstein-barr-virus, treponema pallidum, parvovirus B 19, cytomegalie virus, mycoplasma pneumonia, coxsackivirus type B1-B6, chlamydia pneumonia and toxoplasmosis. Tuberculosis was ruled out by mendel-mantoux skin test, microscopy of Ziehl-Nelson stained urine samples, PCR of a urine and liquor sample, and chest x-ray.

The boy was transferred to the intensive care unit and hyponatremia was balanced within the next 12 h with intravenous application of NaCl 5.85%. Over the course of the next 5 h until his serum sodium was above 125 mmol/l, he had repeated convulsions for up to 30 minutes. Due to status epilepticus and marked agitation, intubation and artificial ventilation was required.

A second CT scan on the next day showed a novel hemorrhage of the left adrenal as well and bleeding in the right pararenal region had enlarged (Figure 3- see Additional file 3). A CT scan of the head was normal, cortisol was low and the adrenals were unresponsive to the Synacten test. We started treatment with hydrocortisone, fludrocortisone and 10 days later with phenprocoumon.

Under this treatment the boy improved clinically within two days and was extubated. As well, laboratory signs of hepatitis normalized within the next three weeks and hemoglobin remained in normal range after blood transfusion. The C-reactive protein normalized within 10 days and antibiotic treatment was stopped as no other clinical or laboratory signs of infection were present.

We continued to monitor the patients condition. As shown in table 1, lupus anticoagulant and cardiolipin antibodies remained positive. He did not develop any other autoimmune diseases (Table 1). The boy's parents and his brother were negative for lupus anticoagulant and cardiolipin antibodies.

## Discussion

The antiphospholipid syndrome is a rare disease in childhood, significantly related to thrombotic events. However, in our patient, initial acute intraabdominal hemorrhage was the major finding. Due to the large hematoma, no clear anatomical association to retroperitoneal organs could be established. Although acute bleeding may sometimes be associated with this syndrome, in this case, it was obvious that microthrombotic events in the adrenal veins led to secondary hemorrhage, because coagulation tests were never critically changed (Tab. 1). Additionally, in the ultrasounds during the first week, the left adrenal gland was slightly enlarged. This was an indirect sign of thrombosis, which proceeded the hemorrhaging 10 days later. In most reported cases, adrenal failure has been linked to thrombotic complications of veins leading to bilateral hemorrhage [4,6,7]. There are five other reported cases of antiphospholipid syndrome and adrenal failure in childhood [6,7,9-11]. However, the clinical presentation is very variable (see Tab. 2- see Additional file 4) and gastrointestinal symptoms are not always present.

The acute adrenal failure 10 days after admission, documented by the novel hemorrhage of the left adrenal gland in the second CT scan, was followed by acute hyponatremia caused by bilateral adrenal insufficiency. Infarction or hemorrhage of the adrenal is a rare but known complication of the antiphospholipid syndrome. Signs of adrenal insufficiency appear when more than 90% of the adrenal cortex is destroyed. The possibility of infectious or autoimmune adrenalitis, by far the most common cause of adrenal insufficiency, was excluded from consideration.

In our patient, the onset of organ insufficiency occured very rapidly as cortisol and serum sodium were still normal six and two days before, respectively. Thus, the failure of the complete adrenal function has to be attributed to the second hemorrhage. In addition, acute hyponatremia was accompanied by hemolytic anemia and elevated transaminases. Recent studies suggest that these symptoms are autoimmune mediated, however, the pathogenic mechanism is not well understood [2-4]. One different hypothesis is due to the unique nature of the vascular anatomy of the adrenal glands since they are rich with arteial supply (3 arteries with up to 60 branches) but limited with venous drainage by a single vein. This however may predispose thrombotic events.

Another complication in our patient was the development of renal symptoms combined with hematuria and proteinuria. Although renal function and blood pressure was appropriate, β-2-microglobulin, a marker for tubular injury, increased during the phase of hematuria (Table 1). In a recent study Nochy et al. reported 16 patients with APS. All of them had biopsy proven vascular nephropathy caused by small vessel vaso-occlusive lesions [8]. Therefore, it is possible that involvement of the small renal vessels also played a role in our patient.

This case demonstrates that APS in childhood may present with a diverse and severe set of symptoms. Adrenal hemorrhage due to thrombosis should be considered as a manifestation of the antiphospholipid syndrome. These patients must be closely monitored because consecutive thrombosis with secondary hemorrhage may rapidly cause adrenal insufficiency.

## Competing Interests

The author(s) declare that they have no competing interests.

## Supplementary Material

Additional File 1 Table 2 Click here for file

Additional File 2 Figure 1: scanned ultrasound photography Click here for file

Additional File 3 Figure 2: scanned ultrasound photography Click here for file

Additional File 4 Figure 3: scanned photography of computer tomography Click here for file
