# Supplementary material for: Adrenal failure followed by status epilepticus and hemolytic anemia in primary antiphospholipid syndrome
Source: Thromb J. 2005 Apr 18;3:6. doi: 10.1186/1477-9560-3-6 (PMC1087888; doi:10.1186/1477-9560-3-6)
Supplement: Additional File 1 — Table 2 [file 1477-9560-3-6-S1.doc]

**Table 2** Demogrpahic, clinical, and immunologic characteristics of 5 patients with adrenal imvolvement associated with antiphospholipid antibodies

**Patient Sex Age (yrs) Clinical manifestation Hyponatremia Hyperkalemia anti-CL Lupus anti-DNA-Ab**

**(Ref.) of adrenal insufficiency anticoagulant**

9 f 10 Tiredness, Hypotension, irritability + IgG, M, A + -

10 f 15 Altered mental status, lethargy, Dehydration IgG, M -

6 m 10 Abdominal pain, vomiting, fever, confusion + normal + +

7 m 3 days Hypotonia + - -

11 m 11 Anorexia, weight loss, hypotension, vomiting + + + -

own m 14 abdominal pain, fever, cough + normal IgG + -

case
