# Supplementary figures and images for: Adrenal failure followed by status epilepticus and hemolytic anemia in primary antiphospholipid syndrome
Source: Thromb J. 2005 Apr 18;3:6. doi: 10.1186/1477-9560-3-6 (PMC1087888; doi:10.1186/1477-9560-3-6)

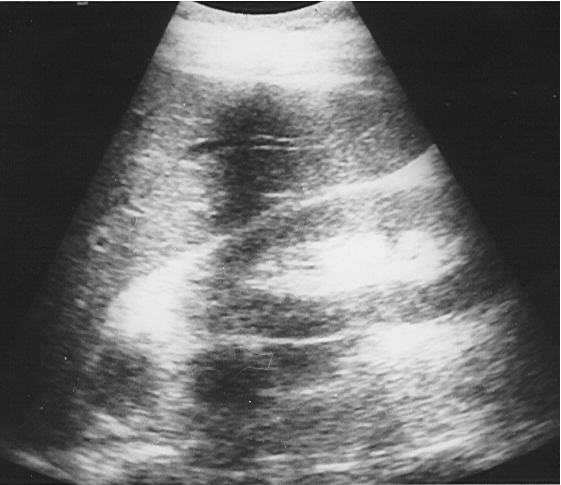

Supplement: Additional File 2 — Figure 1: scanned ultrasound photography [file 1477-9560-3-6-S2.doc]

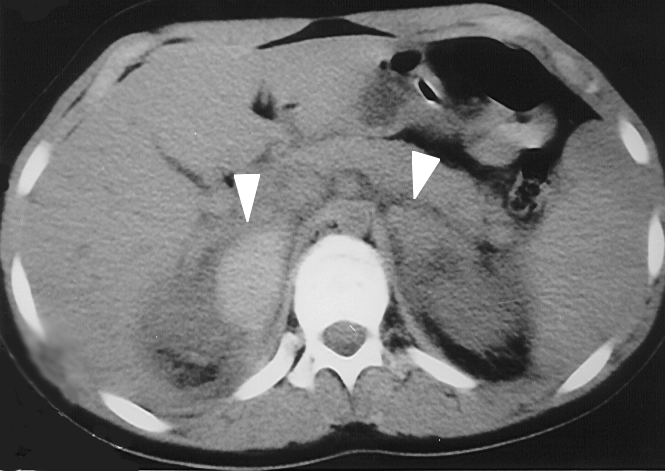

Supplement: Additional File 3 — Figure 2: scanned ultrasound photography [file 1477-9560-3-6-S3.doc]

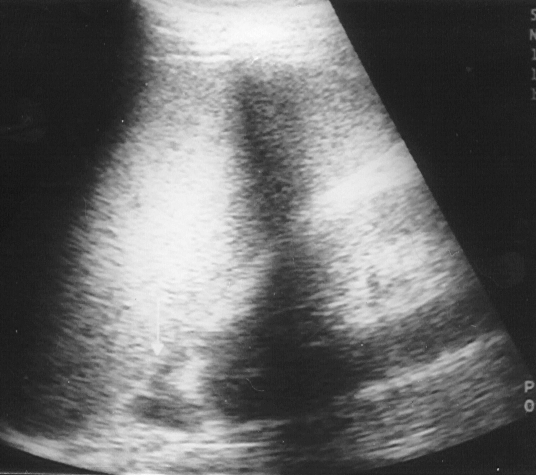

Supplement: Additional File 4 — Figure 3: scanned photography of computer tomography [file 1477-9560-3-6-S4.doc]
